# Is it too late now to say we’re sorry? Examining anxiety contagion and crisis communication strategies using machine learning

**DOI:** 10.1371/journal.pone.0274539

**Published:** 2022-09-12

**Authors:** Dritjon Gruda, Adegboyega Ojo

**Affiliations:** 1 School of Business, National University of Ireland Maynooth, Maynooth, Ireland; 2 Department of Applied Informatics in Management, Faculty of Management and Economics, Gdansk University of Technology, Gdansk, Poland; Shandong University of Science and Technology, CHINA

## Abstract

In this paper, we explore the role of perceived emotions and crisis communication strategies via organizational computer-mediated communication in predicting public anxiety, the default crisis emotion. We use a machine-learning approach to detect and predict anxiety scores in organizational crisis announcements on social media and the public’s responses to these posts. We also control for emotional and language tones in organizational crisis responses using a separate machine learning algorithm. Perceived organizational anxiety positively influences public anxiety, confirming the occurrence of emotional contagion from the organization to the public. Crisis response strategies moderated this relationship, so that responsibility acknowledgment lowered public anxiety the most. We argue that by accounting for emotions expressed in organizational crisis responses, organizations may be able to better predict and manage public emotions.

## Introduction

Whenever organizations face a crisis event–an unexpected event that can severely alter the public’s perception of the organization and impair organizational reputation–they attempt to mitigate and take control of a crisis narrative using various crisis communication strategies [1]. One of the most well-known theories in that regard is Situational Crisis Communication Theory [SCCT; 2]. Based on SCCT, previous work has found that public reaction to an organizational crisis is largely driven by the type of crisis and the preventability of the crisis [3]. Several studies have also considered the role of behaviors and emotion in the public’s reactions to an organizational crisis [2, 4, 5]. However, previous approaches to understanding the role of emotion in crisis communication have largely ignored the link between emotions expressed by the organization (i.e. perceived organizational emotion) and the public’s emotional reactions in response to organizational crises [6].

To address this gap, we propose and empirically examine an approach that integrates SCCT and emotional contagion to better understand how perceived emotions in organizational responses can influence the public’s expressed emotional responses [7–11]. We argue that emotional contagion—“the tendency to automatically mimic and synchronize expressions, vocalizations, postures, and movements with those of another person’s and, consequently, to converge emotionally” [12, p. 5]–may assist in understanding how emotions expressed by the organization as part of their crisis communication response are transferred to the public [13]. Put differently, the public’s emotional experiences, in response to a negative stimulus such as an organizational crisis [9, 13] may be triggered by the emotion conveyed in the organization’s crisis response, in addition to the organization’s respective crisis response strategy.

We do this by re-examining public emotional reactions to crisis events and specific crisis communication strategies outlined in an available dataset on organizational crisis communication and public reactions [14]. We focus on the transfer of the default crisis emotion from the organization in crisis to the public, namely anxiety [4, 15]. The anxiety expressed in an organization’s crisis communication responses (i.e., perceived organizational anxiety) and public anxiety are determined by a machine learning approach [16]. In addition, we control for a set of previously identified crisis emotions (i.e., anger, fear, anger, sadness) and language tones (i.e., confident, tentative, analytical) in organizations’ crisis communication responses. Finally, we contribute to the crisis literature by taking an emotion-based perspective on understanding organizational crisis responses, answering calls for more research on the role of emotions in organizational crisis responses [3] and emotional contagion theory in crisis communication in particular [13].

### Emotional contagion and crisis communication

According to Barsade, et al. [17], emotional contagion describes the transfer and sharing of discrete emotions from one person to another, a process which is largely driven “via subconscious and conscious processes that transpire when people are both elicitors and targets of emotional contagion” (p. 138). Yet, emotional contagion not only impacts the transfer of emotions from individuals to others but also subsequent actions and behaviors by others in response to the transferred emotions. In sum, emotional contagion describes a social influence in which “[person] A has power over [person] B to the extent that [A] can get B to do something that B would otherwise not do” [18, pp. 202–203]. In this case, the power of person A over person B is affective. Importantly, affect describes a term comprising all aspects of subjective feelings, including emotions, moods, and subjective feelings in general [19]. As stated by Gruda, et al. [10], “[i]f person A is successful in transferring their emotions to person B, person B non-consciously imitates the communicated affect, which leads to convergence in both interaction partners’ emotions” (p. 2).

While most works have examined the role and importance of emotional contagion in explaining the transfer of emotions from individuals to others in face-to-face interactions [for a review see 17], several studies have shown that emotional contagion also can occur in online interactions, for example from leaders to followers [10, 11]. Interestingly, in line with previous research on face-to-face interactions, negative emotions are more likely to spread via CMC on social media platforms (in particular) compared to positive emotions [7, 20, 21]. It is this phenomenon that makes emotional contagion particularly suited to better understanding the spread of emotions, such as anxiety, during crisis circumstances.

One of the most known theories of crisis response strategies SCCT [22] states that perceived crisis responsibility attributed to an organization determines which response strategy is most appropriate, with the ultimate goal of reducing reputational damage. For example, on the one hand, certain crises, such as natural disasters, constitute low attribution of crisis responsibility and low reputational threat [1]; in such cases, denial response strategies are recommended (incl. simple denial, shifting the blame or attacking the accuser). On the other hand, crises with high attribution of responsibility and reputational threat are best managed by implementing corrective action and mortification strategies, to minimize reputational damage. Coombs’ work is largely based on crisis responsibility as the core driver of determining the most effective post-crisis communication strategy. Yet, Coombs and colleagues’ work remains a cognition-based perspective and is limited in its understanding of “the role of emotions in how organizations and publics respond to crises” [15]. Put differently, most previous works studied emotions as an outcome that needs to be managed, as opposed to predictors of publics’ emotional reactions [for an exception see 23].

An alternative framework, based on SCCT, addresses the role of emotions in crisis communication, namely the Integrated Crisis Mapping model [ICM; 4, 5]. This model goes a step further in that it focuses on the experience of four discrete emotions (i.e., anxiety, anger, fear, and sadness) experienced by key stakeholders in crisis situations and maps these four emotions onto crisis types. However, as stated by Kim and Cameron [23], “the ICM and SCCT both [continue to] emphasize the public’s emotions are shaped by crisis type and crisis situations” (p. 829). Hence, both models argue that the experience of emotions by the public is a function of a cognitive evaluation of crisis type, crisis responsibility, organizational engagement, and coping resources [13]. This definition of the cognition-to-emotion approach is quite narrow and does not account for the possible transfer of emotions from the organization to the public, as is the case during emotional contagion [7, 9].

In the presented paper, we argue that crisis perception is not simply a function of environmental stimuli and objective facts, but rather relies on the interpretation of the (emotional) stimulus. Hence, we argue for the use of a systematic approach from an emotion-based perspective, given that “emotions are one of the anchors of the publics’ interpretation of the unfolding and evolving events” [4, p. 268]. In particular, we examine how emotion and tone in organization responses can transfer and shape the public’s emotional reaction to crises, in addition to the role of crisis communication response strategies.

Based on emotional contagion, we argue that it is likely that emotions in crisis communication are transferred from the organization to the public, oftentimes automatically and unconsciously [9]. Hence, the public’s emotions are not simply triggered by a cognitive evaluation of the crisis type and situation [13], the foundation of SCCT and ICM but also by the transferred emotion. We argue that this transfer of emotions is particularly likely to occur in times of crisis, as crisis emotions such as anxiety, fear, anger, and sadness [as outlined in the ICM; 4] elicit stronger and more far-reaching emotional responses than positive events [9]. In fact, social media [24] or other forms of many-to-many communication systems, seems to accelerate the transfer of emotions between users and can elicit collective emotions as well [25], leading to a collective experience of a discrete emotion [e.g., anger; 21].

Regarding the present study, we decided to focus on one discrete emotion, namely anxiety. Anxiety is an individual’s physio-psychological reaction to specific stimuli (threat) from the external environment and acts as a precursor of distress [26] and general ill-being when experienced over longer periods. And although anxiety is one of four discrete crisis emotions, anxiety serves as the default emotion in crises, regardless of crisis type [15]. Thus, when faced with “an imminent, specific and overpowering threat” [15: 3] such as the consequences of an organizational crisis, individuals experience anxiety and begin to search for and implement quick crisis resolutions to reduce the experienced anxiety [5]. We argue that the public is most likely to experience anxiety in any case, given the initial uncertainty on how to cope with the crisis and how the organization might continue to manage and react to the fallout of the crisis [4]. Hence, we would expect that the perceived anxiety in organizations’ crisis response communications (e.g., a respective, crisis-relevant post from the organization on social media) is positively associated with public anxiety, as expressed in public interactions with the organization (e.g., comments in response to an organization’s crisis communication). We hypothesize the following:

*H1*: *Perceived organizational anxiety is positively related to the public*’*s experienced anxiety.*

### The role of crisis response strategies

While our primary premise of this paper is the focus on the role of emotions and emotional contagion in crisis communication, we acknowledg that attributions are “a negotiated feature of crisis management, and, therefore, subject to social influence” [27: 352]. Hence, crisis response strategies remain an important factor in understanding the public’s emotional reactions to crises and constitute a factor organizations can control. Previous work has outlined several typologies, which allow capturing and group organizations’ responses to crises [for a review see 3]. For example, responses usually fall on a continuum of either responsibility evasion (i.e., defensive responses, including defiance and scapegoating) or responsibility acknowledgment (i.e., accommodative strategies, including a full apology, expressions of sympathy, corrective action, and mortification). Responses that fall in the middle of the continuum are referred to as responsibility minimizing [1] or reducing offensiveness (i.e., limited organizational responsibility, including excuses).

Previous research, based on SCCT and focusing on crisis type, has found that strategies of responsibility acknowledgment, such as mortification, are an effective crisis communication strategy and can lower public anger and outrage [28]. The picture is less clear regarding the interaction between experienced public emotions and the respective response strategy [3, 27]. Yet, we would still expect a responsibility acknowledgment strategy to be most beneficial to organizations with regard to crisis communication.

In short, strategies based on responsibility acknowledgment are likely to moderate the relationship between perceived organizational anxiety and public anxiety, and by doing so lower public anxiety. However, given the lack of previous research on the specific link between crisis response strategies and emotional contagion, we examine the moderating differences between responsibility acknowledgment and other strategies (e.g., responsibility minimization and evasion) in an exploratory manner. We hypothesize the following:

*H2*: *Crisis response strategies moderate the relationship between perceived organizational anxiety and public anxiety*, *in that strategies based on responsibility acknowledgment are more likely associated with lower public anxiety in comparison to strategies based on responsibility minimization (H2a) and responsibility evasion (H2b)*.

## Methodology

To test our hypotheses, we base our work on the previously published dataset by [14], which includes 505 public responses in direct reply to 18 corporate crisis announcements, excluding instances in which corporations respond to the public’s posts. Crisis response strategies were coded by three trained graduate students as part of the original study by Ki and Nekmat (2014). This was done by “manually review[ing] every Facebook page that had served in a crisis management capacity. When a page displayed an official company’s statement or message regarding a crisis, the researchers noted the specific crisis response strategy used. This study considered the following six crisis response strategies: ‘denial,’ ‘attack the accuser,’ ‘scapegoating,’ ‘excuse,’ ‘justification,’ and ‘full apology’”(p. 144). Intercoder reliability for crisis response type (ICC = 0.81) and crisis type (= 0.89) was deemed adequate [29].

All responses were in reply to corporate crisis announcements made on Facebook. An example of each organizational crisis post is provided in S1 Table in the S1 File.

We annotated the identified 505 public responses and 18 organization crisis responses using a linguistic analytics-based anxiety detection algorithm [16, 30]. A minimal anonymized data set is available online (https://osf.io/ey8ks). Linguistic-based text analytics exploits information about the syntax and semantics of a language as well as lexicons, to extract important information from textual data, such as personality traits [31] or emotions [e.g., 32]. For instance, Gruda and Ojo [33] have demonstrated that mental health signals can be identified from publicly available Twitter data.

### Measures

#### Perceived organizational and public anxiety

In this work, we apply the state and trait anxiety prediction algorithm described in Gruda and Hasan [16] and Gruda and Hasan [30] to measure perceived organizational and public anxiety in response to various corporate crisis communication strategies. This algorithm is based on a dataset of 600 randomly selected micro-blogs from 10,386 users. These tweets were rated by 604 human raters from the US on perceived state anxiety using the short version of the traditional full state-trait anxiety (STAI) scale [34] composed of 6 items on a four-point scale (1 = “Not at all” and 4 = “Very much”). More information regarding this measure can be found in the Supplementary Materials document.

#### Emotional and language tones expressed in organization responses

De Waele, et al. [6] argued that mixed-valence emotions (e.g., a combination of anger and hope) might positively influence organizational reputation. However, organizations might not be aware of whether their message also reflects additional, unintended, emotions. For example, an organization might come across as both anxious and angry, albeit perceived anger was an unintended emotion expressed in organization crisis communications. Hence, we control for all other relevant crisis emotions (i.e., anger, sadness, and fear) and also control for the language tone of the organization’s responses (i.e., analytical, tentative, and confident). To do so, we employed the IBM Watson Tone Analyser (IBM, 2020). Importantly, the IBM Watson Tone Analyzer does not measure anxiety. Anxiety was measured solely using the developed algorithm by Gruda and Hasan (2019). More information regarding the IBM Watson Tone Analyzer can be found in the Supplementary Information document.

## Results

After accounting for all emotional and language tones in organizational crisis responses, we found that none of the organizational responses included anger or fear. Therefore, these emotional tones were not included in the list of variables. Correlations between variables are presented in Table 1.

**Table 1:** Pairwise correlations of main variables.

Variables	M	SD	(1)	(2)	(3)	(4)	(5)	(6)	(7)	(8)
(1) Public Anxiety	2.36	.38								
(2) Perceived Organizational Anxiety	2.12	.21	0.11*							
(3) Analytical (language tone)	.43	.40	0.09*	0.03						
(4) Tentative (language tone)	.28	.37	0.14***	0.49***	0.39***					
(5) Sadness (emotional tone)	.02	.12	0.00	0.01	0.18***	0.28***				
(6) Confident (language tone)	.05	.18	-0.08	-0.04	-0.06	-0.18***	-0.05			
(7) Responsibility Evasion	.14	.35	-0.03	0.31***	0.33***	0.35***	-0.08	0.19***		
(8) Responsibility Minimization	.17	.37	0.16***	-0.14***	0.47***	0.20***	-0.09*	-0.11**	-0.18***	
(9) Responsibility Acknowledgment	.66	.48	-0.11**	-0.23***	-0.58***	-0.45***	0.14***	-0.19***	-0.57***	-0.62***

Note

***p < .001

**p < .01

*p < .05; n = 505 observations.

To maximize the number of observations per crisis response strategy and to ensure that our dataset is not skewed by crisis strategies with only a limited number of observations, we decided to drop the response strategy denial (i.e., 19 observations). In addition, to avoid overfitting our model, we decided to group similar responses based on prior typologies (Benoit, and Coombs, 2007). Hence, we formed a new categorical variable composed of responsibility evasion by blaming others (i.e., attacking the accuser and scapegoating), responsibility minimization (i.e., excuse), and responsibility acknowledgment strategies (i.e. justification, full apology) to account for crisis communication responses in our models.

Given that the observations (i.e., public responses) in our dataset are nested (i.e., specific to a respective organizational crisis and communication response), we accounted for possible company-specific differences by fitting a multi-level mixed-effects regression model with heteroscedastic robust standard errors. Regression results for both examined hypotheses (H1 and H2) are provided in Table 2 below.

**Table 2:** Interaction of perceived organizational anxiety and crisis response strategy on public anxiety.

	M1	M2	M3
Perceived organizational anxiety (POA)	.23**	.17*	-.37***
	(3.04)	(2.38)	(-6.27)
Responsibility Evasion (Blaming others)	(baseline)	(baseline)	(baseline)
Responsibility Minimization (Excuse)	.21**	.20***	-1.52***
	(3.10)	(3.46)	(-4.44)
Responsibility Acknowledgment (Apology)	.06	.06	-1.13***
	(1.09)	(1.16)	(-6.74)
POA X Responsibility Evasion (Blaming others)			(baseline)
POA X Responsibility Minimization (Excuse)			.77***
			(5.02)
POA X Responsibility Acknowledgment (Apology)			.50***
			(8.49)
Sadness		-.03	0.5
		(-.56)	(.51)
Tentative		.07	-.00
		(1.17))	(-.03)
Analytical		-.02	-.03
		(-.69)	(-.99)
Confident		-.05	-.11*
		(-1.15)	(-2.45)
Constant	1.81***	1.9***	3.20***
	(9.92)	(11.46)	(17.38)
Wald χ^2^	17.19***	421.83***	1006.44***
n	505	505	505

Note: z-scores in parentheses

*** *p* < .001

** *p* < .01

* *p* < .05

^†^ *p* < .10

Firstly, results show a positive association between perceived organizational anxiety and public anxiety (Model 1, Table 1: *b* = .23, *SE* = .08, z = 3.04, *p* = .002). Results remain similar when accounting for additional emotions and tones expressed in the organizations’ crisis responses (Model 2, Table 1: *b* = .17, *SE* = .07, z = 2.38, *p* = .017). Hence, it seems that anxiety expressed in organizations’ crisis responses positively influences public anxiety. This provides support for H1.

**Fig 1:**
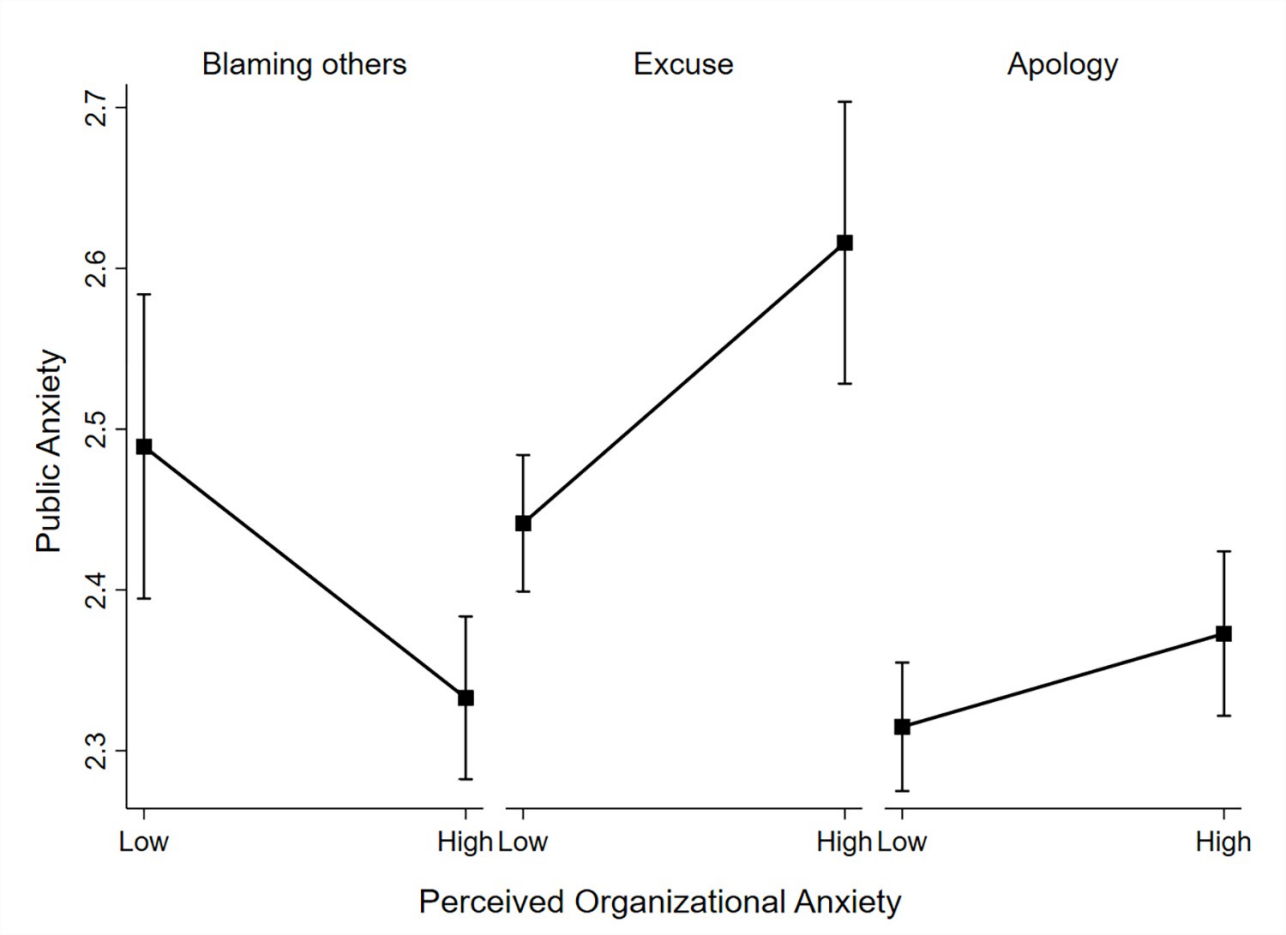
Interaction between perceived organizational anxiety and crisis response strategies on public anxiety.

Results from the two-way interaction (Model 3, Table 2) show that compared to responsibility minimization strategies (i.e., excuse; Model 3, Table 2: *b* = .77, *SE* = .15, *z* = 5.02, *p* = .000), responsibility acknowledgment strategies were less positively associated with public anxiety (Model 3, Table 2: *b* = .50, *SE* = .06, *z* = 8.49, *p* = .000). However, in order to understand this interaction better, we graphed results per group (see Fig 1).

Simple slope analysis of the interaction (+/- 1 SD, Fig 1) across the three different crisis response strategies shows that both responsibility minimization (i.e., excuse: simple slope = .41, *SE* = .12, *z* = 3.52, *p* = .000) and responsibility acknowledgment (i.e., full apology or justification: simple slope = .14, *SE* = .04, *z* = 3.05, *p* = .002) were associated with a positive increase in public anxiety. The increase in public anxiety was lower in the case of crisis acknowledgments strategies. This provides support for H2a. However, responsibility evasion strategies (i.e., attack the accuser, scapegoating) seemed to be negatively associated with public anxiety (simple slope = -.37, *SE* = .06, *z* = -6.27, *p* = .000). Therefore, we do not find sufficient evidence in support of H2b.

In sum, based on the results, we find that when organizations used responsibility minimization or responsibility acknowledgment strategies, higher levels of perceived organizational anxiety—compared to lower levels of perceived organizational anxiety–led to higher levels of public anxiety. In contrast, when organizations used responsibility evasion strategies, higher levels of perceived organizational anxiety–compared to lower levels of perceived organizational anxiety–led to lower levels of public anxiety. Hence, in our sample, anxiety expressed in organizational crisis responses increased public anxiety when the respective organization used responsibility minimization or acknowledgment strategies, but decreased public anxiety when the organization used responsibility evasion strategies (e.g., blaming others). Next, we interpret and discuss these results further in line with previous literature.

## Discussion

Social media is an emerging tool for disseminating information quickly and publicly and can play a vital role in crisis management purposes [35]. And crisis management on social media platforms can be both beneficial if communication is handled appropriately [36] and detrimental to organizational reputation if mishandled [1]. In the presented work, we argue that perceived anxiety in organizational crisis communication predicts public anxiety. In doing so, this study finds support for this relationship and makes several contributions to the crisis communication and emotional contagion literature [13].

Firstly, addressing calls for more research on the role of emotional contagion in crisis communication [3], we find that anxiety is expressed in social media posts and responses and seems to be transferrable even when institutions, namely organizations, communicate to the public. This is likely due to the personification of organizations and brands, which results in customers and the public alike expecting human-like qualities when corresponding with these organizations. This supports previous work which found that emotions are easily transferred on social media between leaders to followers via social media interactions [10]. And given that emotions expressed on social media, in particular negative emotions, can trigger the viral sharing of content [10], we emphasize that the emotional experiences of the public should not be ignored in favor of less complex categorizations such as positive or negative valence.

Secondly, we find that crisis response strategy moderates the relationship between perceived organizational anxiety and public anxiety.

We hypothesized that in comparison to responsibility minimization strategies (i.e., excuses), corrective crisis response strategies, such as full apology, result in lower public anxiety. And in both cases, higher perceived organizational anxiety was associated with higher public anxiety, confirming that emotional contagion had taken place. In contrast, higher perceived organizational anxiety in the case of responsibility evasion, or blaming others (i.e., attacking the accuser and scapegoating) seemed to be associated with lower public anxiety. This finding is interesting because defensive crisis response strategies seem contrary to reducing risk in crisis communication. However, it is important to keep in mind that public anxiety is a response to the level of perceived organizational anxiety. Thus, defensive strategies might not lower public anxiety as such, but they might lower public anxiety when perceived organizational anxiety is high compared to when perceived organizational anxiety is low. In addition, concerning defensive strategies, public anxiety decreases as perceived organizational anxiety increases, while for more accommodative strategies, public anxiety increases when perceived organizational anxiety increases. Yet, a closer look at Fig 1 shows that in the case of apology strategies, levels of public anxiety overall (regardless of perceived organizational anxiety) are still lower than levels of public anxiety in the case of defensive strategies. Suggesting that defensive strategies might be as effective as more accommodative strategies is thus not correct.

## Implications

The importance of the study of the transfer of anxiety from individuals to others [9] or from leaders to followers in organizations [10] has been well established. However, we argue that the study of the transfer of anxiety from organizations to the public is underdeveloped and warrents further investigation. We based this argument on a) the lack of research conducted on public anxiety as a crisis emotion in response to organizational announcements on social media and b) because the experience of anxiety can have destructive consequences [37] that go well beyond harming organizational reputation, but rather can carry personal mental health consequences for customers and the public alike, including depleted self-regulation, and increased and prolonged emotional exhaustion [38].

Crisis can emerge quickly and organizations need to be prepared to not only face such crises but also to effectively communicate to their customers and the public alike, to restore trust in the organization and lower public anxiety. This communication primarily takes place online as organizations address the public, and subsequently must manage the potential fall-out from communicating heightened levels of anxiety, which, in combination with the respective crisis response strategy, can increase public anxiety as a response. The COVID-19 pandemic has raised the importance of online communication between organizations and the public, changing the way people communicate, collaborate, and work [39, 40]. Due to the particular circumstances of the COVID-19 pandemic, namely forced social distancing, the use of social platforms of communication has increased dramatically as most communication moved online. In the present paper, we found that the degree of perceived organizational anxiety is important in determining the public response, and investigated the moderating role of crisis response strategies on the relationship between organizational and public anxiety. However, due to the small sample size, we could not test whether crisis type also matters in predicting the degree of public anxiety. Hence, while this study serves to provide preliminary insights into the relationship between perceived organizational anxiety and public anxiety, organizations would be remiss to not consider crisis response strategies and perceived organizational anxiety in light of the particular crisis type they might be facing. In that sense, we suggest that the role of crisis context should not be ignored and must be considered when considering the transfer of anxiety from organizations to the public. This is particularly crucial during a crisis (or crisis-like circumstances), in which individuals oftentimes feel more vulnerable and anxious, which can easily result in defensive strategies on the individual level including distancing oneself from the threat (i.e., the organization) and spreading increased negative emotions about the organization as a coping mechanism [41].

Finally, in this paper, we have outlined the usefulness of applying a developed machine learning approach to measure and differentiate public perception of different crises and crisis responses [e.g., the COVID-19 pandemic, 40] going forward. Hence, our work shows that organizations could use such computerized methods to a) retrospectively account for previous corporate crises and develop improved crisis response policies and strategies and b) measure public anxiety in response to crisis responses in real-time as well [10, 33]. The application possibilities of the presented machine learning algorithm can help steer corporations in the right direction and potentially reduce the possible loss and fallout of a crisis to a minimum. For example, organizations could establish a crisis early warning system and prepare the organization, personnel, and set aside funding for the company to respond to the crisis efficiently and effectively [35].

In sum, organizations must understand that their social media communication to the public matters, particularly so during a crisis. Organizations must learn to take responsibility and not attempt to resolve a crisis by the use of responsibility minimization or evasion strategies [42]. In this respect, our study demonstrates the need for organizations not only to focus on communicating quickly to the public (a factor not explored in this study) but also to communicate strategically by understanding the critical role of expressed anxiety and carefully chosen crisis response strategy. Specifically, organizations should place more importance on the role of social media communication when attempting to address a crisis.

## Limitations and future research

The presented research has certain limitations. Firstly, the sample size of this study is limited. However, we limited the sample size purposefully to public replies in direct response to a respective corporate crisis announcement. Future research could expand on the findings reported in this research work by examining whether the found results would still hold when customers are the first to shed light on an occurring crisis, basing future results on a larger sample size.

Secondly, the presented study focuses on one social media platform, namely Facebook. Different social media platforms have different user groups with potentially different reactions to a company’s crisis response [1]. This could result in different public responses to crises. Future researchers could extend the presented research by examining and comparing public anxiety in response to corporate crisis communication strategies on several social media platforms.

## Conclusion

Based on previous research [14], we explored the role of emotional contagion and crisis communication strategies in predicting public anxiety, and the default crisis emotion [15]. We found that higher perceived organizational anxiety is associated with higher levels of public anxiety, confirming emotional contagion as an underlying phenomenon. In addition, and in support of SCCT, we find that crisis communication response strategies moderate the relationship between organizational and public anxiety, in that defensive communication strategies (i.e., attacking the accuser or scapegoating) can in fact lower public anxiety. We argue that these findings warrant future research, potentially investigating the role of crisis type in addition to crisis communication strategies. Finally, we argue that by accounting for emotions expressed in organizational crisis responses, organizations may be able to better predict and manage public emotions.

## Supporting information

S1 File (DOCX)
