# Supplementary material for: Is it too late now to say we’re sorry? Examining anxiety contagion and crisis communication strategies using machine learning
Source: PLoS One. 2022 Sep 12;17(9):e0274539. doi: 10.1371/journal.pone.0274539 (PMC9467322; doi:10.1371/journal.pone.0274539)
Supplement: S1 File — (DOCX) [file pone.0274539.s001.docx]

**Supplementary Materials**

**Methodology**

***Emotional and language tones expressed in organization responses:*** The IBM Watson Tone Analyzer service is an industrial-strength linguistic analytics tool that relies on foundations in psycholinguistics and machine-learning models to infer personality characteristics, thinking styles, emotions, and intrinsic values from written text. The Tone analyzer model was trained on a set of pre-processed 96,000 customer-care-related tweets of selected brands which were collected between June and August 2016 (IBM, 2020). The trained model can detect tones classified as analytical, tentative, joy, sadness, confident, anger, and fear. The different tone classes relevant to this study (i.e., crisis emotions) are described as follows (Steffens et al., 2020): analytical – the text expresses a person’s reasoning and analytical attitude about things; tentative – expresses some degree of inhibition; sadness – expresses the feeling of loss and disadvantage, quiet or less energetic; and confident – expresses some degree of certainty; anger – expresses injustice, conflict, humiliation, negligence or betrayer and a direct attack on the target; fear – expresses danger. For our analysis, we used the ToneAnalyzerV3 package from the IBM Watson Cloud libraries to generate the tone profiles for each element of the pair. The perceived degree of each tone element range from 0 to 1.

**S1 Table**

*Examples of Crisis Communication Category and Announcement Examples*

| **Communication Strategy** | **Sample Example** |
| --- | --- |
| Attack the accuser | *“Playing responsibly is important to Mattel. Over the past months, we’ve been talking to Greenpeace regarding paper-sourcing. As you may have heard, they’ve taken an inflammatory approach despite the open channels of communication we’ve established. You can learn more about our corporate responsibility efforts & packaging improvements here: […]”.* |
| Denial | *“Unknown parties pretending to be Procter & Gamble have been sending unsolicited emails to individuals falsely offering jobs that require the cashing of checks and then forwarding the proceeds via Western Union to others. P&G wants you to know that those emails are NOT authentic, that P&G does not require anyone to engage in such activities, and performing check cashing services in this manner may be illegal.”* |
| Excuse | *“We have received some questions regarding the discontinuation of our debit card rewards program. As a result of changes in Federal law, we are ending our debit card rewards program. Before reaching this decision, we surveyed members to evaluate options and members told us they would prefer to keep other benefits such as free checking. To learn more about the new federal law, how we reached our decision and what this means to members, please click below...”* |
| Full apology | *“We’re issuing a voluntary recall of several toy Frisbees that may shatter when punctured, which may be harmful to the pet & pet parent. Go to the below site for information on how to determine if your Frisbee is affected by the recall. Bring the affected Frisbees to your closest PetSmart store for a full refund. If you have questions about this recall, call PetSmart’s Customer Care team at […].”* |
| Justification | *"The proposed merger will not result in job losses for existing U.S. wireless call center employees of T-Mobile and AT&T. After closing the proposed merger with TMobile USA, AT&T will bring back 5,000 wireless call center jobs to the United States that today are outsourced to other countries. This would be the largest return of jobs by any U.S. company since 2008. […]”.* |
| Scapegoating | *“Today [our company] launched an investigation into deforestation allegations. While [our company] does not contract directly with Sinar Mas/APP, we have directed our packaging suppliers to stop sourcing pulp from them as we investigate the allegations. You can learn more here: […].”* |

Note: Adopted from Ki and Nekmat (2014)**.**
